# Acute Effects of Substitution, and Addition, of Carbohydrates and Fat to Protein on Gastric Emptying, Blood Glucose, Gut Hormones, Appetite, and Energy Intake

**DOI:** 10.3390/nu10101451

**Published:** 2018-10-07

**Authors:** Caroline Giezenaar, Kylie Lange, Trygve Hausken, Karen L. Jones, Michael Horowitz, Ian Chapman, Stijn Soenen

**Affiliations:** 1 Adelaide Medical School and National Health and Medical Research Council of Australia (NHMRC) Centre of Research Excellence (CRE) in Translating Nutritional Science to Good Health, The University of Adelaide, Adelaide 5000, Australia; caroline.giezenaar@adelaide.edu.au (C.G.); kylie.lange@adelaide.edu.au (K.L.); karen.jones@adelaide.edu.au (K.L.J.); michael.horowitz@adelaide.edu.au (M.H.); ian.chapman@adelaide.edu.au (I.C.); 2 Department of Medicine, Haukeland University Hospital, 5021 Bergen, Norway; trygve.hausken@helse-bergen.no; 3 Royal Adelaide Hospital, Adelaide 5000, Australia

**Keywords:** whey protein, gastric emptying, gut hormones, blood glucose, appetite

## Abstract

Whey protein, when ingested on its own, load-dependently slows gastric emptying and stimulates gut hormone concentrations in healthy young men. The aim of this study was to determine the effects of substitution, and addition, of carbohydrate (dextrose) and fat (olive oil) to whey protein. In randomized, double-blind order, 13 healthy young men (age: 23 ± 1 years, body mass index: 24 ± 1 kg/m^2^) ingested a control drink (450 mL; ~2 kcal/‘control’) or iso-volumetric drinks containing protein/carbohydrate/fat: (i) 14 g/28 g/12.4 g (280 kcal/‘M_280′_), (ii) 70 g/28 g/12.4 g (504kcal/‘M_504′_), and (iii) 70 g/0 g/0 g (280 kcal/‘P_280′_), on 4 separate study days. Gastric emptying (*n* = 11, 3D-ultrasonography), blood glucose, plasma insulin, ghrelin, cholecystokinin (CCK) and glucagon-like peptide-1 (GLP-1) concentrations (0–180 min), appetite (visual analogue scales), and ad-libitum buffet-meal energy intake (180–210 min) were determined. Substitution of protein with carbohydrate and fat was associated with faster gastric emptying (lower 50% emptying time (T50)), reduced suppression of ghrelin, and stimulation of GLP-1 (all *P* < 0.001); while the addition of carbohydrate and fat to protein did not affect gastric emptying or gut hormone responses significantly. Total energy intake (i.e., drink plus meal) was greater after all caloric drinks than control (*P* < 0.001). In conclusion, substitution of whey protein with dextrose and olive oil accelerated gastric emptying. Higher protein content of a mixed macronutrient drink increased gut hormone and insulin responses.

## 1. Introduction

The gastrointestinal tract plays a central role in determining appetite, energy intake, and postprandial glycemic excursions [1]. The stomach regulates the rate of delivery of ingested nutrients to the small intestine to optimize their digestion and absorption. When nutrients enter the small intestine they generate feedback signals that slow gastric emptying and suppress appetite, via both neural and hormonal mechanisms, which includes stimulation of cholecystokinin (CCK) and glucagon-like peptide (GLP-1) and suppression of ghrelin [2]. The lowering of postprandial blood glucose concentrations by GLP-1 is attributed in part to slowing of gastric emptying [3].

Protein-rich supplements and diets are used widely for weight loss purposes, based on the rationale that intake of dietary protein has muscle sparing, and greater satiating effects, than carbohydrate and fat [4,5]. The satiating effect of ingested protein may be attributed, at least in part, to slowing gastric emptying. Our studies in healthy younger men have shown that whey protein, when infused intraduodenally, increases pyloric, and decreases antral and duodenal, motility, a pattern of contractions associated with retardation of gastric emptying [6,7]. Oral ingestion of whey protein alone load-dependently slows gastric emptying rates and increases plasma insulin, ghrelin, CCK, and GLP-1 concentrations in healthy young men [7]. The caloric rate of emptying of protein, carbohydrate, and fat, assessed by magnetic resonance imaging (MRI), may be similar when ingested alone [8], but protein and other macronutrients are seldom ingested in isolation. It is, accordingly, important to determine their effects on gastric emptying and other parameters when ingested in combination. There is evidence that protein evokes higher postprandial plasma CCK and GLP-1 responses and slows gastric emptying, as assessed by the acetaminophen absorption test, more than the other macronutrients, when ingested in a mixed macronutrient combination (yoghurt, 400 kcal, energy percent protein/carbohydrates/fat: 58/14/28% vs. 19/47/34%) [9]. Ingestion of whey protein slowed gastric emptying and reduced postprandial blood glucose concentrations in type 2 diabetes patients [10]. Gastric emptying half time, as determined by a ^13^CO_2_ breath test, was comparable between a high- vs. low-fat breakfast (pancakes, ~780 kcal, energy percent protein/carbohydrates/fat: ~10/30/60% vs. ~6/70/24%) [11].

The aim of this study was to determine the acute effects of substitution, and addition, of carbohydrate and fat to whey protein liquid supplements on gastric emptying, blood glucose and plasma insulin, ghrelin, CCK, and GLP-1 concentrations, perceptions of appetite and gastrointestinal symptoms, and ad libitum energy intake at a buffet meal in healthy young men. We hypothesized that the equi-energetic replacement of protein by carbohydrate and fat would result in relatively faster gastric emptying, whereas the addition of carbohydrate and fat (and hence energy) to protein would be associated with slower gastric emptying and increased suppression of ghrelin and stimulation of CCK and GLP-1, compared to control.

## 2. Materials and Methods

### 2.1. Subjects

Thirteen healthy men aged between 18 and 30 years (Mean ± standard error of mean (SEM): Age: 23 ± 1 years; body weight: 78 ± 2 kg; height: 1.79 ± 0.02 m; body mass index (BMI): 24 ± 1 kg/m^2^) were recruited by advertisement. Exclusion criteria were alcohol abuse, use of illicit substances, smoking, use of medications known to potentially affect energy intake, appetite, or gastrointestinal motor function), significant gastrointestinal symptoms (abdominal pain, heartburn, diarrhea, or constipation), gastrointestinal surgery (apart from uncomplicated appendectomy), diabetes, gallbladder or pancreatic disease, known lactose intolerance or food allergies, low plasma ferritin levels or blood donation in the 12 weeks prior to the study, and failing to comprehend the study protocol. Subjects provided written informed consent. The study was approved by the Royal Adelaide Hospital Human Research Ethics Committee (140407), conducted in accordance with the Declaration of Helsinki, and registered as a clinical trial with the Australian New Zealand Clinical Trial Registry (www.anzctr.org.au; ACTRN12614000846628). 

### 2.2. Protocol

This study had a randomized (using the method of randomly permuted blocks; www.randomization.com), double-blind, cross-over design. Subjects were studied on 4 occasions, each separated by 3–14 days, to determine the effects of substitution, and addition, of carbohydrate and fat to whey protein liquid supplements (Table 1) compared to control on gastric emptying, gut hormones, perceptions of appetite and gastrointestinal symptoms, and energy intake. In ‘M_280_′, 56 g, of the 70 g pure protein load (‘P_280_′) was substituted for fat and carbohydrates, and in M_504_, the same amount of fat and carbohydrates was added.

Drinks were matched for taste, served in a covered cup, and prepared by dissolving whey-protein isolate (Fonterra Co-Operative Group Ltd., Palmerston North, New Zealand) and dextrose (glucose), and homogenizing olive oil (Bertolli Australia Pty Ltd., Unilever Australasia, Sydney, NSW, Australia) in varying volumes of demineralized water and diet lime cordial (Bickford’s Australia Pty Ltd., Salisbury South, SA, Australia).

Subjects consumed a standardized evening meal (beef lasagna (McCain Foods Pty Ltd., Wendouree, VIC, Australia), ~591 kcal) on the night before each study day at 19.00 h. They were furthermore instructed to fast overnight from solids and liquids and to refrain from strenuous physical activity.

In each subject, blood sampling from an intravenous cannula, ultrasound measurements of gastric volume, and appetite ratings were obtained immediately before (during fasting), and at regular time intervals after ingestion of each drink until 180 min. Subjects were seated in an upright position on a wooden chair after removal of all metal objects, instructed to consume the drink within 2 min, and presented with a standard, cold, buffet-style meal in excess of what they were expected to consume (180 min). The meal had a total energy content of 2457 kcal, with 19% protein, 50% carbohydrates, and 31% fat. The subjects were allowed to eat for 30 min (180–210 min) until comfortably full. They were in a room by themselves whilst eating to limit external distractions.

### 2.3. Measurements

#### 2.3.1. Gastric Emptying by 3D Ultrasonography

Gastric volume was measured by a Logiq™ 9 ultrasound system (GE Healthcare Technologies, Sydney, NSW, Australia) with TruScan Architecture including a 3D sensor, equipped with a 3.5C broad spectrum 2.5–4 MHz convex transducer [12]. The stomach was scanned using a continuous translational movement along its long axis (~10 s) at 0, 5, 15, 30, 45, 60, 75, 90, 105, 120, 135, 150, 165, and 180 min. The raw data (original scan planes) were transferred for 3D reconstruction and volume estimation using EchoPAC-3D software (GE Vingmed Sound, Horten, Norway). When ultrasound images lacked sufficient clarity to determine the volume of the stomach at some time points, data were imputed by linear interpolation [13]. In two subjects, the quality of ultrasound stomach images was insufficient to determine gastric emptying during one or more entire study days, and all data related to gastric emptying in these subjects were, therefore, excluded from analysis.

Intragastric retentions from 0–60 min ‘early’ and 60–180 min ‘late’ were calculated as total gastric volume minus baseline ‘fasting’ gastric volume at each time point, expressed as percentage of the maximal gastric volume (100%), i.e., volume of the ingested drink. The time at which 50% of the drink had emptied from the stomach (50% gastric emptying time; T50; min) and ‘complete’ gastric emptying time (100% gastric emptying time; T100; min) were calculated for all conditions. ‘Complete emptying’ was defined as the time when the residual volume of the drink in the stomach was ≤5%, and set to 180 min when the residual volume at 180 min was ≥5%. Rate of gastric emptying was calculated as the mean of rates of emptying (kcal/min) during each 15-min interval, respectively, of the ‘early’ (0–60 min) and ‘late’ (60–180 min) phases [13].

#### 2.3.2. Blood Glucose and Plasma Insulin, Ghrelin, CCK, and GLP-1 Concentrations

Blood samples were collected into ice-chilled, EDTA-coated tubes at 0, 5, 15, 30, 45, 60, 90, 120, 150, and 180 min. No inhibitors were added [14]. Blood glucose concentrations (millimoles per liter) were determined immediately after collection by the glucose oxidase method using a portable glucometer (Optium Xceed, Abbott Laboratories, Doncaster, VIC, Australia). 

Plasma was obtained by centrifugation for 15 min at 3200 rpm (1032 g) at 4 °C. Plasma samples were stored at −80 °C. Plasma total insulin concentrations (milliunits per liter) were measured by enzyme-linked immunosorbent assay (ELISA) immunoassay (10–1113; Mercodia, Uppsala, Sweden), with a detection limit of 1.0 mU/L and intra- and inter-assay coefficients of variation of 3.0% and 6.8%. Plasma total ghrelin concentrations (picograms per milliliter) were measured using a radioimmunoassay (RIA; NEX388; Perkin Elmer, Boston, MA, USA) [15], with a detection limit of 40 pg/mL and intra- and inter-assay coefficients of variation of 5.1% and 10.1%. Plasma CCK-8 concentrations (picomoles per liter) were measured by RIA (C2581; Sigma Chemical, St. Louis, MO, USA) [15], with a detection limit of 1 pmol/L and intra- and inter-assay coefficients of variation of 8.1% and 11.5%. Plasma total GLP-1 concentrations (picomoles per liter) were measured by RIA (GLPIT-36HK; Millipore, Billerica, MA, USA) [15], with a detection limit of 3 pmol/L and intra—and inter—assay coefficients of variation of 2.7% and 7.1%.

Peak/nadir and time to peak/nadir concentrations for blood glucose, plasma insulin, ghrelin, CCK, and GLP-1 were calculated for the caloric drink conditions.

#### 2.3.3. Perceptions of Appetite and Gastrointestinal Symptoms

Hunger, fullness, prospective consumption, desire to eat, nausea, and bloating were determined by a visual analogue scale (VAS) questionnaire at 0, 5, 15, 30, 45, 60, 75, 90, 105, 120, 135, 150, 165, 180, and 210 min [16].

#### 2.3.4. Energy Intake

Energy and protein, carbohydrate, and fat intakes were determined at a buffet-style meal. The meal consisted of palatable food items, including sliced bread, margarine, ham, chicken, cheese, mayonnaise, fruit, fruit salad, yoghurt, custard, orange juice, iced coffee, and water [13]. Absolute energy intake (kcal), both as the intake at the buffet meal and as the cumulative energy intake (buffet meal plus preload drink), and the percentage suppression/change energy intake at the buffet meal (expressed as % of energy intake of the control day) by a given protein load compared to control, were calculated.

### 2.4. Data and Statistical Analyses

Statistical analyses were performed using SPSS software (version 22; IBM, Armonk, NY, USA). On the basis of our previous work [13], with an observed within-subjects SD of 31 min for gastric emptying half time, we calculated that 13 subjects would allow detection of a within-groups difference between treatments for T50 of 35 min, with power equal to 0.8 and alpha equal to 0.05. Differences between study conditions for gastric emptying, glucose, hormones, appetite, and energy intake were determined using one-way repeated-measures ANOVA, with the treatment as the within-subject factor, and Bonferroni post hoc comparisons. Interaction effects of time by treatment for glucose, hormones, and appetite were determined using a two-way repeated measures ANOVA, with treatment and time as the within-subject factors. Within-subject correlations were determined by using a general linear model with a fixed slope and random intercept [17]. Area under the curve (AUC) for gastric emptying, glucose, hormones, and appetite were calculated from baseline to 60 min (early phase), and 60 to 180 min (late phase), using the trapezoidal rule. Peak/nadir and time to peak/nadir hunger, fullness, prospective food consumption, desire to eat, nausea, and bloating were calculated for all conditions. Assumptions of normality were verified for all outcomes before statistical analysis. Statistical significance was accepted at *P* < 0.05. Data in the text and tables are presented as mean values ± SD, and data in the figures are presented as mean values ± SEMs.

## 3. Results

Baseline gastric volumes (mean ± SD: 37 ± 3 mL), blood glucose (5.3 ± 0.4 mmol/L), plasma insulin (4.9 ± 3.0 mU/L), ghrelin (1779 ± 851 pg/mL), CCK (1.3 ± 0.4 pmol/L), and GLP-1 concentrations (20 ± 7.3 pmol/L), hunger (46 ± 26 mm), fullness (4 ± 3 mm), prospective food consumption (56 ± 15 mm), desire to eat (50 ± 17 mm), nausea (6 ± 6 mm), and bloating (6 ± 4 mm), were not different between the four study days.

### 3.1. Gastric Emptying

**Figure 1:**
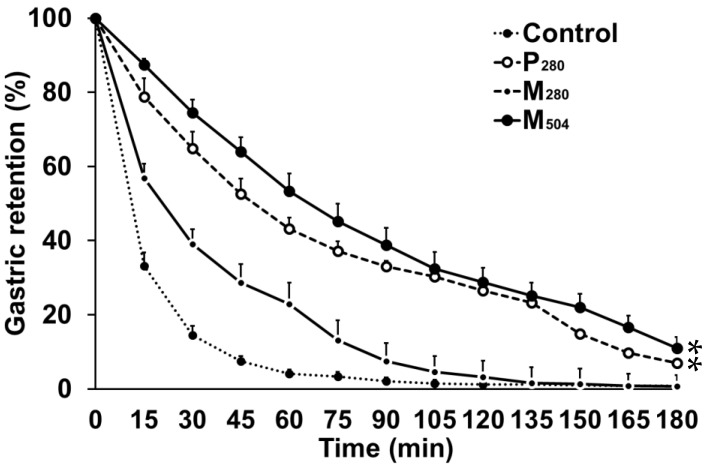
Intra-gastric retention (%; *n* = 11 healthy young men) of a control drink (450 mL; ~2 kcal/‘control’) or iso-volumetric drinks containing protein/carbohydrate/fat: (i) 14g/28 g/12.4 g (280 kcal/‘M_280_′), (ii) 70 g/28 g/12.4 g(504kcal/‘M_504_′), or (iii) 70 g/0 g/0 g(280 kcal/‘P_280_′). Effects of drink-condition were determined by repeated-measures ANOVA with post hoc Bonferroni corrections. * *P* < 0.05, post hoc tests: P_280_ and M_504_ gastric emptying was slower than M_280_ and control. Data presented as mean (± standard error of mean (SEM)).

The M_280_ and control drink emptied in a non-linear pattern, whereas the pattern of emptying of M_504_ and P_280_ was more linear (Figure 1). Gastric emptying of all caloric drinks was slower compared to control (*P* < 0.05). Gastric emptying of P_280_ and M_504_ were slower (*P* < 0.001), compared to M_280_, with greater 50% and 100% gastric emptying times after both P_280_ and M_504_ compared to M_280_ (*P* < 0.001, Table 2). Gastric emptying of M_504_ was not significantly slower than P_280_, with the stomach taking 21% more time to empty 50%, (70 vs. 58 min), but only 4% more time to empty 100% (176 vs. 169 min) after the addition of fat and carbohydrate. 

### 3.2. Blood Glucose and Plasma Gut Hormone Concentrations

#### 3.2.1. Glucose

**Figure 2:**
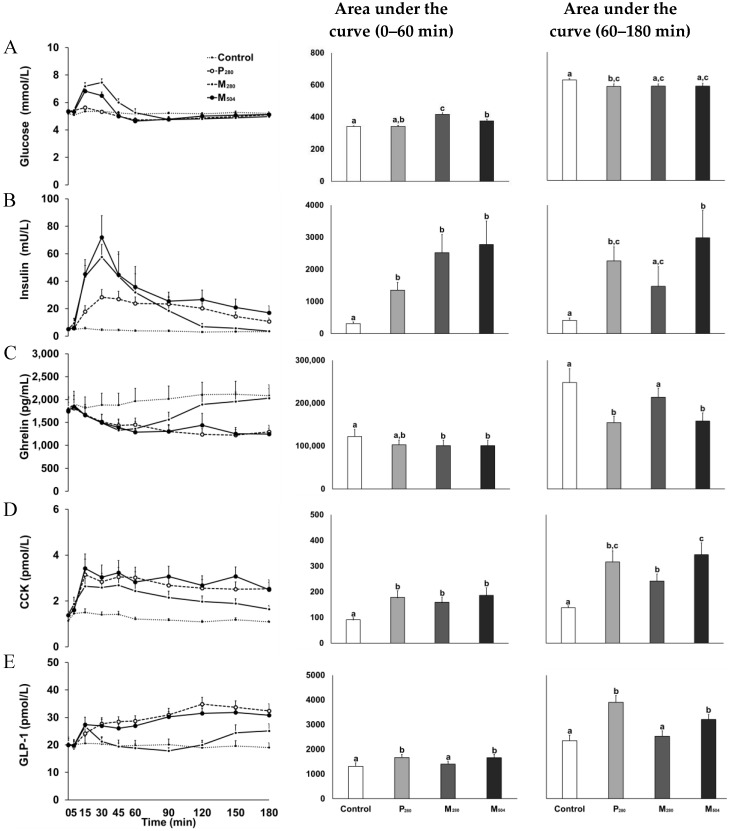
Blood glucose (A; mmol/L), plasma insulin (B; mU/L), ghrelin (C; pg/mL), cholecystokinin (D; CCK, pmol/L), and glucagon-like polypeptide-1 (E; GLP-1, pmol/L) concentrations (*n* = 13 healthy young men) after drinks containing either: (i) 70 g whey protein (280 kcal; ‘P_280′_); (ii) 14 g protein, 28 g carbohydrate, 12.4 g fat (280 kcal; ‘M_280′_); (iii) 70 g protein, 28 g carbohydrate, 12.4 g fat (504 kcal; ‘M_504′_); or (iv) a control drink (~2 kcal). Effects of time, drink-condition, and the interaction effect of time by drink-condition were determined by repeated-measures ANOVA with post hoc Bonferroni corrections. There was an interaction effect of time by drink-condition for blood glucose (*P* < 0.001), insulin (*P* < 0.001), ghrelin (*P* < 0.001), CCK (*P* < 0.001), and GLP-1 concentrations (*P* < 0.001). ^a,b,c^, *P* < 0.05, post hoc test: Different letter indicates significant difference (e.g., for glucose AUC_0–60 min_: M_280_ (c) higher than control (a), P_280_ (a,b) and M_504_ (b); M_504_ (b) higher than control (a); P_280_ (a,b) not different from control (a) or M_504_ (b)) in area under the curve (AUC, ‘early phase’: 0–60 min and ‘late phase’: 60–180 min) between drink-conditions. Data presented as mean (± standard error of mean (SEM)).

Glucose increased after both mixed-macronutrient drinks and returned to baseline at ~60 min (i.e., M_280_ and M_504_; interaction effect of time by drink-condition: *P* < 0.001, Figure 2). Peak glucose concentrations after M_280_ (7.9 ± 0.6 mmol/L) and M_504_ (7.0 ± 0.7 mmol/L) were higher (*P* = 0.001) than after P_280_ (5.7 ± 0.4 mmol/L) and control (5.6 ± 0.2 mmol/L) and AUC_0–60min_ concentrations higher than control (*P* < 0.001). Early concentrations (AUC_0–60min_) were higher after M_280_ than M_504_ and P_280_ (*P* < 0.001), while late concentrations (AUC_60–180min_) were less after P_280_ than control (*P* = 0.026).

#### 3.2.2. Insulin

Insulin increased after all caloric drinks (P_280_, M_280_, and M_504_; interaction effect of time by drink-condition: *P* < 0.001, Figure 2); AUC_0-60min_ (*P* = 0.005) and peak concentrations (*P* = 0.001) were higher compared to control (Table 2). M_280_ and M_504_ evoked a rapid increase in insulin concentrations, with higher AUC_60-180min_ concentrations after M_504_ than M_280_ (*P* = 0.004), while insulin remained elevated after M_504_ and P_280_, with 180-min concentrations being higher after P_280_ compared to M_280_ (*P* < 0.009).

#### 3.2.3. Ghrelin

Plasma ghrelin concentrations decreased after all caloric drinks; concentrations returned to baseline at ~120 min after M_280_, and remained below baseline until the buffet meal at 180 min after P_280_ and M_504_ (interaction effect of time by drink-condition: *P* < 0.001, Figure 2). Nadir ghrelin concentrations following P_280_ and M_504_ were lower than control (*P* = 0.003, Table 2) and remained suppressed, with AUC_60–180min_ and 180-min concentrations being lower compared to M_280_ and control (*P* < 0.001). 

#### 3.2.4. CCK

CCK increased promptly after all caloric drinks (interaction effect of time by drink-condition: *P* < 0.001, Figure 2). Peak and AUC_0–60min_ CCK concentrations after P_280_, M_280_, and M_504_ were higher than control (*P* < 0.001, Table 2) and remained elevated, with AUC_60–180min_ and 180-min concentrations being higher than control, as well as after M_504_ compared to M_280_ (*P* < 0.001).

#### 3.2.5. GLP-1

GLP-1 increased after all caloric drinks (interaction effect of time by drink-condition: *P* < 0.001). In the first 15 min, GLP-1 concentrations increased comparably after M_280_ and M_504_ (Figure 2). Peak GLP-1 concentrations after P_280_ (mean at ~87 min) and M_504_ (mean at 115 min) were higher than control (*P* < 0.001, Table 3) and remained elevated, with higher AUC_0–60min,_ AUC_60–180min_, and 180-min concentrations, immediately before buffet-meal intake, compared to both M_280_ and control (*P* < 0.001). 180-min GLP-1 concentrations were also higher after P_280_ compared to M_280_ (*P* < 0.001).

### 3.3. Perceptions of Appetite and Gastrointestinal Symptoms

**Figure 3:**
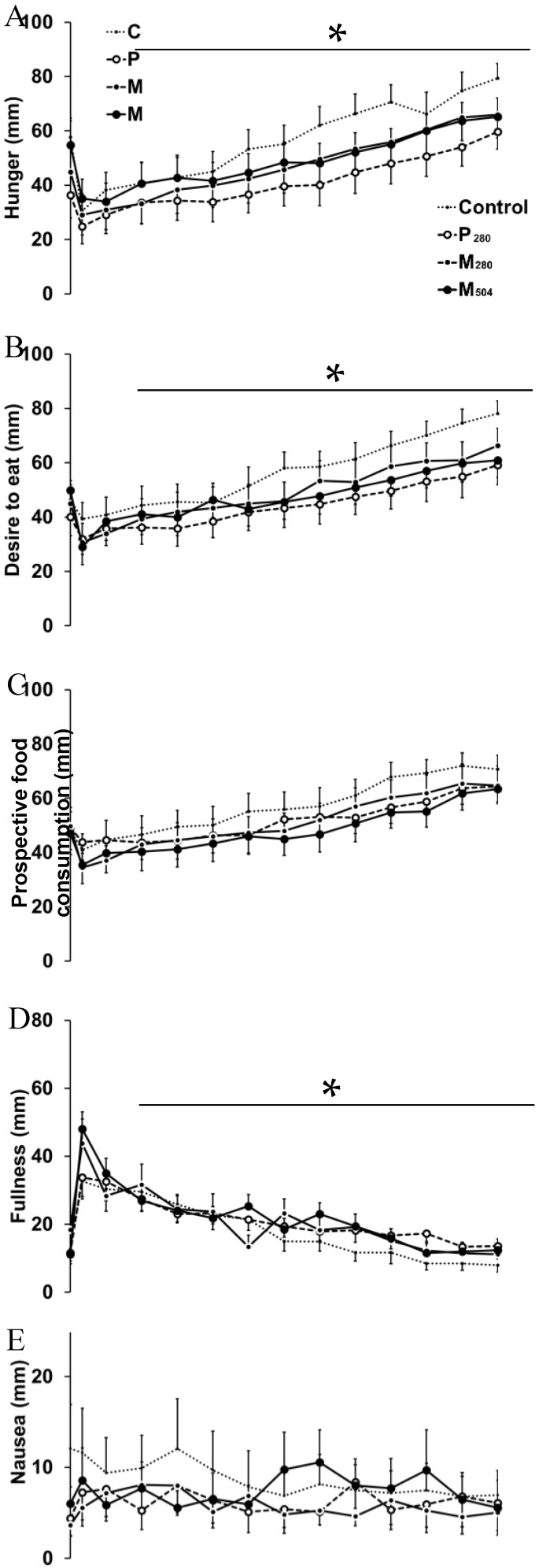

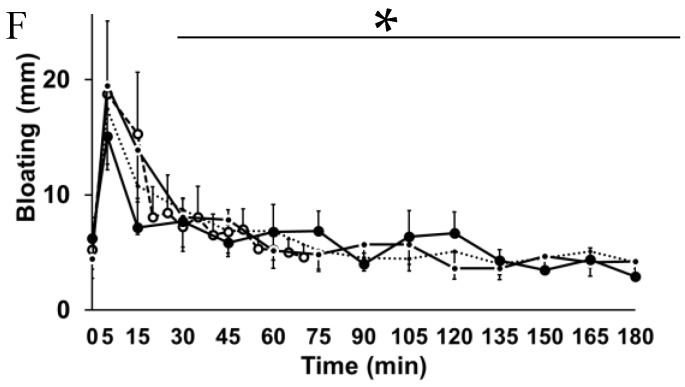
Visual analogue score (VAS, mm) of hunger (**A**), desire to eat (**B**), prospective food consumption (**C**), fullness (**D**), nausea (**E**), and bloating (**F**; *n* = 13 healthy young men), after ingestion of a control drink (450 mL; ~2 kcal/‘control’) or iso-volumetric drinks containing protein/carbohydrate/fat: (i) 14 g/28 g/12.4 g (280 kcal/‘M_280_′); (ii) 70 g/28 g/12.4 g (504 kcal/‘M_504_′); or (iii) 70 g/0 g/0 g (280 kcal/‘P_280_′). Effects of time, drink-condition, and the interaction effect of time by drink-condition were determined by repeated-measures ANOVA with post hoc Bonferroni corrections. * Effect of time was significant for hunger (*P* < 0.001), desire to eat (*P* < 0.001), fullness (*P* < 0.001), and bloating (*P* = 0.023). Data presented as mean (± standard error of mean (SEM)).

Hunger (mean decrease of four study visits: 25 ± 12 mm, time to nadir: 34 ± 30 min, *P* < 0.001), and desire to eat (19 ± 13 mm, 28 ± 25 min, *P* < 0.001) decreased after drink ingestion and increased thereafter to ratings higher than baseline immediately before the buffet meal (180 min). AUC_0–60min_ hunger (control: 2615 ± 440 mm, P_280_: 2049 ± 460 mm, M_280_: 2238 ± 491 mm, M_504_: 2559 ± 452 mm; *P* = 0.021) and desire to eat (control: 2809 ± 411 mm, P_280_: 2323 ± 385 mm, M_280_: 2473 ± 432 mm, M_504_: 2555 ± 434 mm; *P* = 0.021) were lower after P_280_ than control, but not than M_280_ or M_504_. Fullness (37 ± 16 mm, 35 ± 25 min, *P* < 0.001) and bloating (18 ± 20 mm, 21 ± 20 min, *P* = 0.023) increased after the drink and returned to baseline thereafter (Figure 3). Prospective food consumption (*P* = 0.14) and nausea (*P* = 0.27) did not change over time.

### 3.4. Energy Intake

**Figure 4:**
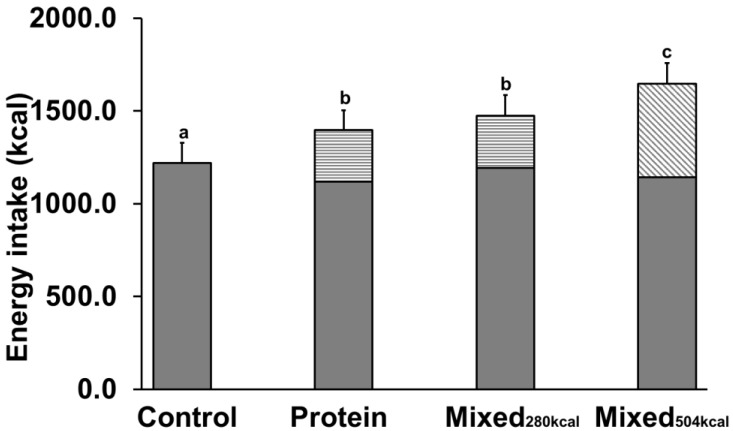
Energy intake (*n* = 13 healthy young men) at the buffet meal (kcal; black shading) after ingestion of a control drink (450 mL; ~2 kcal/‘control’) or iso-volumetric drinks containing protein/carbohydrate/fat: (i) 14 g/28 g/12.4 g (280 kcal/‘M_280_′); (ii) 70 g/28 g/12.4 g (504 kcal/‘M_504_′); or (iii) 70 g/0 g/0 g (280 kcal/‘P_280_′ - energy content of the drink as the striped part of each bar). Effects of drink-condition were determined by repeated-measures ANOVA with post hoc Bonferroni corrections. ^a,b,c^ *P* < 0.05, post hoc tests: Different letter indicates difference in total energy intake (meal plus drink), which was higher after P_280_, M_280_, and M_504_ compared to control, and higher after M_504_ compared to P_280_ and M_280_. Data presented as mean (± standard error of mean (SEM)).

Ad libitum energy (Figure 4) and protein (P), carbohydrate (C), and fat (F) intakes were comparable between study days (mean of four study days: Energy intake 1168 ± 382 kcal *P* = 0.16, P 21 ± 4.3% *P* = 0.16, C 47 ± 11% *P* = 0.96, F 32 ± 7% *P* = 0.99). As a result, total energy intake (i.e., drink plus meal) was greater after all caloric drinks than control (*P* < 0.001), and greater after M_504_ than P_280_ and M_280_ (*P* < 0.05).

### 3.5. Correlations between Gastric Retention, Hormones, and Energy Intake

AUC_60–180min_ concentrations of GLP-1 were, within subjects, negatively (*r* = −0.33, *P* = 0.038), and perceptions of hunger and desire to eat and concentrations of ghrelin positively (range of *r* = 0.33 to 0.66, *P* < 0.05), correlated with energy intake.

AUC_0–60min_ perceptions of hunger, desire to eat and prospective food consumption, and plasma gut hormones, but not blood glucose concentrations were, within subjects, associated with gastric retention; i.e., lower plasma ghrelin, perceptions of hunger, desire to eat, and prospective food consumption (range of *r* = −0.39 to −0.42, *P* < 0.05) and higher insulin, CCK, and GLP-1 concentrations (range of *r* = 0.52 to 0.65, *P* < 0.01) correlated with higher gastric retention. AUC_60–180min_ concentrations of ghrelin were, within subjects, negatively (*r* = −0.74, *P* < 0.001) correlated, and insulin, CCK, and GLP-1 positively (range of *r* = 0.73 to 0.76, *P* < 0.001), correlated with gastric retention.

Plasma concentrations of insulin were, within subjects, associated with blood glucose concentrations (AUC_0–60min_: *r* = 0.48, *P* = 0.002; AUC_60–180min_: *r* = −0.34, *P* = 0.029), and with plasma GLP-1 concentrations (AUC_60–180min_: *r* = 0.054, *P* < 0.001). Plasma insulin concentrations at 180 min were, within subjects, associated with plasma GLP-1 concentrations at 180 min (*r* = 0.38, *P* = 0.017).

## 4. Discussion

This study determined the acute effects of substitution, and adding, of carbohydrate and fat to protein on gastric emptying, glucose, gut hormones, appetite, and energy intake in healthy young men. Equi-energetic substitution of 224 kcal fat (112 kcal) and carbohydrate (112 kcal) for whey protein in the 280-kcal drinks (M_280_ vs. P_280_) resulted in faster gastric emptying, particularly during the early phase, greater increases in glucose concentrations, less prolonged suppression of ghrelin, a tendency for a smaller increase in CCK, and a marked abbreviation of the duration of the GLP-1 response. Thus, whey protein slowed gastric emptying more than an equi-energetic combination of protein, fat and carbohydrate under these study conditions. As both CCK [18,19] and GLP-1 [20,21] slow gastric emptying, the lesser increases in their blood concentrations after fat and carbohydrate than protein may, in part, account for the faster gastric emptying after the mixed macronutrient drink. The lower increases in CCK and GLP-1 after M_280_ may be a result of the emptying of fat and protein/carbohydrates in different phases. The drinks in this study were mixed for ~45 min until immediately before their ingestion and the macronutrients were evenly mixed at the time of ingestion. As the subjects were seated during the study, however, it is possible that the fat (olive oil; 9 kcal/g) separated from the protein/carbohydrate solution (4 kcal/g) after ingestion of the M_280_ and M_504_ drinks, and emptied from the stomach slower than the aqueous phase by ‘layering’ on the denser aqueous components [22].

The addition of 224 kcal fat (112 kcal) and carbohydrate (112 kcal) to 280 kcal whey protein (M_504_ vs. P_280_) produced a slight, but not significant, further slowing of gastric emptying and had no additional stimulatory effect on CCK and GLP-1, or suppressive effect on ghrelin, but predictably increased glucose and insulin concentrations above those after whey protein alone [23,24]. The lack of additional stimulation of CCK and GLP-1 may be an explanation for the lack of further slowing of gastric emptying, consistent with a greater slowing effect of protein than fat and carbohydrate, and a role for CCK and GLP-1 in mediating this effect. Our results are in line with the report that gastric emptying of a protein-enriched yoghurt preload is slower than that of a carbohydrate-enriched preload, associated with higher plasma CCK and GLP-1 concentrations after the high protein than high carbohydrate preload [9]. Administration of a fat and carbohydrate drink on its own was not part of this study and would be needed to test the possibility that 224 kcal combined fat and carbohydrate, unlike 280 kcal whey protein, has little slowing effect on gastric emptying, which is possible given the reduced slowing effect of fat and carbohydrate compared to equi-caloric whey protein demonstrated in the substitution part of the study. Alternately, the 280 kcal whey-protein drink may have produced near maximal nutrient-induced slowing, with fat and carbohydrate unable to slow emptying much more, even if they would do so if administered without this protein.

Adding protein to a glucose meal increases the insulin response [25,26,27], as with the addition of extra protein to a mixed macronutrient drink in this study (M_504_ vs. M_280_). This effect is particularly strong after ingestion of whey, which has a high content of insulinotropic amino acids [28], when compared to casein [29]. Despite the higher energy content of M_504_ than M_280_, early phase blood glucose concentrations were lower after M_504_ than M_280_. Factors contributing to this difference could include the higher blood insulin concentrations and an increased ratio of whey protein to dextrose, resulting in lower amounts of carbohydrates entering the small intestine, where they are absorbed. A daily meal pattern of three meals, with snacks in between, results in a limited number of hours of true fasting. Postprandial glycemia predominates over fasting blood glucose in contributing to glycated hemoglobin (HbA1c), a risk indicator of developing diabetes-related complications, and a deterioration in postprandial glycemic control precedes any substantial elevation of fasting blood glucose [30]. As such, our finding indicates that increasing the whey protein content of a drink may have postprandial glucose lowering effects and may be a valid nutritional strategy to manage glycaemia in patients with type 2 diabetes.

The postprandial plasma incretin hormone GLP-1 concentrations seemed to be particularly related to the protein content of the drinks; concentrations were higher after M_504_ and P_280_, which had comparable responses from 30 min onwards, than after M_280_. Initial peak GLP-1 concentrations at 15 min after M_280_ and M_504_ were comparable and slightly higher than P_280_. GLP-1 after M_280_ increased from 120 min onwards until before meal intake (180 min) after the transient initial peak, which may be related to the ‘layering’ of the fat component of the drink in the proximal stomach. Plasma CCK and ghrelin concentrations showed a similar pattern, although slightly less pronounced; when compared to M_280_, M_504_ and P_280_ had a more sustained decrease in ghrelin and slightly higher increases in CCK. Consistent with our results, preloads rich in whey [9,31], whey and egg white [32], whey and soy [33], casein [31], and dairy protein [34] resulted in suppressed plasma ghrelin concentrations [9,31,32,33,34]. Protein vs. glucose [9,31], but not fat [35], resulted in increased plasma CCK concentrations, and protein vs. carbohydrate [9,36,37,38] and fat [36,38,39] resulted in larger and more sustained plasma on GLP-1 concentrations in most, but not all [40,41], studies. Protein increased postprandial insulin concentrations more than fat [40].

The use of high protein supplements in young people is widespread, and increasing, in response to greater awareness that ingestion of protein has a muscle sparing effect and greater satiating effects than dietary carbohydrate and fat [4,5], making it attractive when weight control is desired. Before the test meals, more than 90% of all drinks had emptied from the stomach. Although hunger and desire to eat were lower after P_280_ compared to control, there was a substantial effect on ghrelin, CCK, and GLP-1 at the time of meal ingestion, and energy intake was, within subjects, associated with plasma insulin, ghrelin, CCK, and GLP-1 concentrations, and perceptions of hunger in this study, ad libitum energy intake at the buffet meal was not different after the drinks, and, consequently, there was an increase in total energy intake during the caloric-drink study days compared to control. Energy intake at the buffet meal was assessed three hours after drink ingestion to allow for complete emptying of the drinks from the stomach and thus detailed assessment of gastric emptying and gut hormone responses. It has been reported in a systemic review that the optimal timing of a preload before a meal, if suppression of ad libitum food intake at that meal is intended, is much closer to the meal, with a time interval of 30 min or less associated with the greatest subsequent suppression of energy intake. Furthermore, suppression of energy intake is less after a liquid than semi-solid and solid preload [42].

Limitations included the relatively small subject numbers and blood glucose being measured by a glucometer, which is less than optimal. Nevertheless, any errors in glucose measurement would be expected to be uniform between study conditions and the glucose results were clear-cut. Olive oil and dextrose were used as a substitute for, and addition to, whey protein. Therefore, the results of this study are related to these single types of fat and carbohydrate. Furthermore, the nutrients were ingested in liquid form, and, therefore, the results may not translate to the administration of nutrients in solid form. While the drinks were palatable and matched for taste, we did not assess the subjects’ perceptions of taste and/or pleasantness of the drinks. Energy intake at the buffet meal was assessed three hours after drink ingestion at a buffet meal, but not during the remainder of the day. 

## 5. Conclusions

In conclusion, substitution of whey protein with dextrose and olive oil resulted in faster gastric emptying, less suppression of ghrelin, and less stimulation of CCK and GLP-1 concentrations; while adding the dextrose and olive oil to whey protein did not further slow gastric emptying, suppress ghrelin, or increase CCK and GLP-1 responses of the drink. A higher protein content of the mixed macronutrient drinks slowed gastric emptying and evoked stronger ghrelin, CCK, GLP-1, and insulin responses and a lower increase in blood glucose concentrations.

## Figures and Tables

**Table 1:** Energy content and composition of liquid supplements used in study days.

	Control	P_280_	M_280_	M_504_
Energy content (kcal)	2	280	280	504
Whey protein (g, %)	0, 0	70, 100	14, 20	70, 56
Dextrose (g, %)	0, 0	0, 0	28, 40	28, 22
Olive oil (g, %)	0, 0	0, 0	12.4, 40	12.4, 22

**Table 2:** Gastric emptying parameters.

	Control	P_280_	M_280_	M_504_
50% emptying time (T50; min)	12 ± 3 ^a^	58 ± 31 ^b^	23 ± 8 ^c^	70 ± 29 ^b^
100% emptying time (T100; min)	82 ± 47 ^a^	169 ± 17 ^b^	116 ± 28 ^c^	176 ± 14 ^b^
**Gastric retention (AUC)**				
Early phase (0–60 min, % × 60 min^−1^)	27 ± 6 ^a^	67 ± 12 ^b^	47 ± 10 ^c^	76 ± 10 ^b^
Late phase (60–180 min, % × 120 min^−1^)	2 ± 3 ^a^	25 ± 3 ^b^	6 ± 3 ^a^	30 ± 13 ^b^
**Rate of gastric emptying (kcal/min) ^1^**				
Early phase (0–60 min)		2.7 ± 0.9 ^a^	3.9 ± 1.4 ^b^	3.5 ± 2.1 ^b^
Late phase (60–180 min)		0.7 ± 0.3 ^a^	1.2 ± 0.7 ^b^	1.8 ± 0.4 ^c^
**Amount emptied (%)**				
Early phase (0–60 min)	96 ± 4 ^a^	57 ± 19 ^b^	77 ± 9 ^c^	47 ± 16 ^b^
Late phase (60–180 min)	99 ± 2 ^a^	93 ± 10 ^b^	99 ± 2 ^a^	89 ± 10 ^b^

Mean (± standard error of mean (SD)) 50% and 100% emptying time (min), gastric retention (%), rate of gastric emptying (kcal/min), and amount emptied (area under the curve (AUC), %) during the early (0–60 min) and late phase (60–180 min) of gastric emptying (*n* = 11 healthy young men) of a control drink (450 mL; ~2 kcal/‘control’) or iso-volumetric drinks containing protein/carbohydrate/fat: (i) 14 g/28 g/12.4 g (280 kcal/‘M_280_′); (ii) 70 g/28 g/12.4 g (504 kcal/‘M_504_′); or (iii) 70 g/0 g/0 g (280 kcal/‘P_280_′). ^1^ Rate of gastric emptying was calculated as mean of rates of emptying during each 15-min interval, respectively. Main effects of drink-condition were determined by repeated-measures ANOVA with post hoc Bonferroni corrections. ^a,b,c^, *P* < 0.05, post hoc test: Different letter indicates significant difference between drink conditions (e.g., 50% emptying time: P_280_ (b) slower than M_280_ (c), which are slower than control (a), not different from M_504_ (b)).

**Table 3:** Blood glucose and plasma hormone concentrations.

	Control	P_280_	M_280_	M_504_
Peak/nadir concentration				
Glucose	5.6 ± 0.2 ^a^	5.7 ± 0.4 ^a^	7.9 ± 0.6 ^b^	7.0 ± 0.7 ^b^
Insulin	6 ± 3 ^a^	35 ± 22 ^b^	66 ± 54 ^b^	74 ± 58 ^b^
Ghrelin	1653 ± 837 ^a^	1109 ± 366 ^b^	1269 ± 538 ^a,b^	1110 ± 365 ^b^
CCK	1.7 ± 0.6 ^a^	3.8 ± 2.2 ^b^	3.0 ± 1.3 ^b^	4.0 ± 2.2 ^b^
GLP-1	23 ± 8 ^a^	36 ± 9 ^b^	29 ± 12 ^a,b^	34 ± 8 ^b^
180-min concentration				
Glucose	5.2 ± 0.4 ^a^	5.1 ± 0.4 ^a^	5.0 ± 0.4 ^a^	5.1 ± 0.7 ^a^
Insulin	3.2 ± 2.3 ^a^	11 ± 9 ^b^	3.7 ± 2.7 ^a^	17 ± 18 ^a,b^
Ghrelin	2081 ± 871 ^a^	1295 ± 523 ^b^	2029 ± 754 ^a^	1245 ± 459 ^b^
CCK	1.1 ± 0.2 ^a^	2.5 ± 1.4 ^b,c^	1.6 ± 0.6 ^c^	2.5 ± 1.1 ^b^
GLP-1	19 ± 6 ^a^	32 ± 9 ^b^	25 ± 9 ^a,c^	31 ± 7 ^b,c^
Time to peak/nadir				
Glucose	59 ± 56 ^a^	18 ± 31 ^a^	25 ± 7 ^a^	23 ± 10 ^a^
Insulin	17 ± 12 ^a^	71 ± 39 ^b^	28 ± 8 ^a,c^	31 ± 7 ^c^
Ghrelin	55 ± 64 ^a^	128 ± 38 ^b^	44 ± 13 ^a^	116 ± 53 ^b^
CCK	13 ± 17 ^a^	68 ± 51 ^b^	32 ± 28 ^a,b^	68 ± 61 ^b^
GLP-1	65 ± 61 ^a^	124 ± 32 ^b^	87 ± 78 ^a,b^	115 ± 61 ^a,b^

Mean (± standard error of mean (SEM)) peak/nadir and 180-min concentration, and time to peak/nadir (min) of blood glucose (mmol/L), plasma insulin (mU/L), ghrelin (pg/mL), cholecystokinin (CCK, pmol/L), and glucagon-like polypeptide-1 (GLP-1, pmol/L) concentrations (*n* = 13 healthy young men) after drinks containing either: (i) 70 g whey protein (280 kcal; ‘P_280′_); (ii) 14 g protein, 28 g carbohydrate, 12.4 g fat (280 kcal; ‘M_280′_); (iii) 70 g protein, 28 g carbohydrate, 12.4 g fat (504 kcal; ‘M_504′_); or (iv) a control drink (~2 kcal). Main effects of drink-condition were determined by repeated-measures ANOVA with post hoc Bonferroni corrections. ^a,b,c^, *P* < 0.05, post hoc tests: Different letter indicates significant difference between drink-conditions (e.g., for glucose peak concentrations: M_280_ (b) higher than control (a) and P_280_ (a), not different from M_504_ (b)).
